# Management and Outcomes of Urinary Tract Involvement in Cytoreductive Surgery with Hyperthermic Intraperitoneal Chemotherapy (CRS/HIPEC): A Retrospective Cohort Study

**DOI:** 10.3390/medicina61081331

**Published:** 2025-07-23

**Authors:** Feza Karakayali, Melik Kagan Aktas, Erman Aytac, Ugur Sungurtekin, Sezai Demirbas, Mustafa Oncel, Ersin Ozturk, Tahsin Colak, Mehmet Ince, Mustafa Haksal, Safak Coskun, Selman Sokmen

**Affiliations:** 1 Department of Surgery, Baskent University Istanbul Hospital, Istanbul 34662, Türkiye; 2 Department of Surgery, The Ohio State University, Columbus, OH 43210, USA; 3 Gastrointestinal Oncology Unit, Acibadem University Atakent Hospital, Istanbul 34303, Türkiye; 4 Department of Surgery, Pamukkale University, Denizli 20160, Türkiye; 5 Department of Surgery, TOBB University, Ankara 06510, Türkiye; 6 Department of Surgery, Medipol University, Istanbul 34214, Türkiye; 7 Department of Surgery, Bursa Medicana Hospital, Bursa 16110, Türkiye; 8 Department of Surgery, Mersin University, Mersin 33110, Türkiye; 9 Department of Surgery, Yuzuncu Yil Hospital, Ankara 06530, Türkiye; 10 Department of Surgery, Dokuz Eylul University, Izmir 35330, Türkiye

**Keywords:** cytoreductive surgery, HIPEC, urinary tract, oncology, peritoneal carcinomatosis

## Abstract

*Background and Objectives*: The combined use of cytoreductive surgery (CRS) and hyperthermic intraperitoneal chemotherapy (HIPEC) is employed for the treatment of peritoneal carcinomatosis (PC). To achieve optimal cytoreduction, there may be a need for extensive resection and subsequent reconstruction of urologic structures. This study was designed to evaluate the outcomes of urinary tract resection or repair performed in CRS/HIPEC in terms of operative and oncological outcomes. *Materials and Methods:* After institutional review board approval, data from 550 consecutive patients who underwent the CRS/HIPEC procedure from January 2007 to July 2018 at six university hospitals was retrieved from prospectively maintained databases. Data from patients who had a concomitant curative resection and reconstruction of the bladder, ureter, or kidney during the CRS/HIPEC procedure were analyzed retrospectively. *Results*: A total of 50 out of 550 patients had undergone resection with a repair of the urinary tract due to tumor invasion or iatrogenic injury. Postoperative (within 30 days) urologic complications were observed in 9 of the 50 patients. It was found that having a peritoneal cancer index (PCI) equal to or greater than 20 (*p* < 0.009) was the sole significant risk factor associated with the occurrence of early urinary complications. Survival time post CRS/HIPEC treatment did not significantly differ between patients with and without urologic complications (median overall survival: 23 vs. 27 months, *p* = 0.683). *Conclusions*: Despite urinary tract issues during CRS/HIPEC for PC, including a PCI over 20 and potential complications from resection or repair, the procedure still offers significant survival benefits.

## 1. Introduction

Peritoneal carcinomatosis (PC) is a condition marked by the presence of cancer cells on the peritoneal surface, commonly occurring in advanced stages of gastrointestinal and gynecological cancers as well as primary peritoneal malignancies. For certain individuals, cytoreductive surgery (CRS) and hyperthermic intraperitoneal chemotherapy (HIPEC) are employed as a comprehensive and definitive treatment approach [1]. CRS/HIPEC was first introduced by Spratt et al. in 1980 and subsequently popularized by Sugarbaker et al. [2,3]. The introduction of CRS and HIPEC represents a synergistic approach, combining radical surgery with regional chemotherapy [4]. CRS aims to remove all visible tumor nodules within the intra-abdominal cavity, achieving complete cytoreduction by resecting all peritoneal surfaces with observable disease and removing any organs affected by PC [5,6]. The peritoneum, which could be acknowledged as an organ, is deemed a resectable structure in the CRS phase of the procedure. The extent of resection is used to evaluate residual disease through the completeness of the cytoreduction score (CC). Depending on the severity of PC, achieving complete CRS often involves performing an omentectomy and multiple peritonectomies. Furthermore, this may include the resection of visceral organs in the gastrointestinal or genitourinary systems [6,7]. HIPEC targets the elimination of residual microscopic cancer cells. By administering chemotherapy directly into the peritoneal cavity the procedure enables the delivery of a more concentrated dose to peritoneal disease, minimizing systemic toxicity. Additionally, mild hyperthermia enhances the cytotoxic effects of the drugs and improves their penetration into tumor nodules [4,6]. The studies demonstrated diversity in treatment protocols, particularly with the timing of the procedure, the methodology of administration, and the choice of therapeutic agent(s) [8]. As a result of this circumstance, even after numerous years of development, there is a lack of standardization in HIPEC protocols. These protocols exhibit not only variations in the types of drugs used but also notable variability in drug dosages. Nevertheless, there has been a lack of exploration of technical variations in comparative trials. Two commonly utilized schedules involve oxaliplatin with or without irinotecan, administered concurrently with intravenous 5-fluorouracil and folinic acid, and mitomycin-C, either alone or in conjunction with other drugs [9]. Numerous randomized trials investigating CRS and HIPEC have consistently revealed a survival advantage compared to approaches involving palliation and systemic chemotherapy alone [10,11,12]. Various studies have highlighted that achieving complete cytoreduction is significantly linked to extended disease-free and overall survival (OS), establishing it as the foremost prognostic and predictive factor [13,14]. Prognostic features like the CC and the peritoneal cancer index (PCI) have demonstrated their influence on survival outcomes following CRS and HIPEC, which were specifically analyzed in our study [15,16,17,18,19]. Postoperative complications predominantly hinge on the degree of peritoneal dissemination and the corresponding extent of cytoreduction [20]. It is not rare for the urinary tract to be implicated, particularly following prior surgical explorations that have revealed retroperitoneal structures [5,21,22]. Consequently, extensive urologic resection and reconstruction may be essential to attaining optimal cytoreduction. Genitourinary structures can be affected either through direct tumor invasion or iatrogenic injury during cytoreduction procedures [21]. There are few documented reports on how resected urinary tract involvement may affect the outcomes of patients undergoing CRS/HIPEC [5,21]. Major debulking surgeries, especially those focused on gynecological or colorectal malignancies in the pelvic area, pose a constant risk of lower urinary tract injury. This heightened risk is attributed to the close anatomical proximity and embryonic association between the reproductive, intestinal, and lower urinary tracts [23,24]. Furthermore, the distortion of anatomy and the need for extensive surgery in individuals with gynecological, gastric, and colorectal malignancies that extend to the peritoneum can lead to an increased likelihood of injury. Also, in the course of complex procedures like CRS and HIPEC, it is essential to perform extensive resections and lymphadenectomies before exposing internal organs and structures to hyperthermic perfusion. During such procedures there is a significant risk of ureters experiencing partial devascularization or direct damage, whether it be mechanical, chemical, or thermal [25]. Patients who necessitate urological resection as part of cytoreduction are linked to notable postoperative morbidity and mortality, likely attributed to an overall more extensive cytoreduction [6]. The potential link between urinary tract involvement and more aggressive biological behavior and its subsequent influence on long-term survival is currently under investigation [22]. Individuals with PC and urologic involvement should undergo an assessment at a specialized peritoneal surface malignancy center, and treatment decisions should be tailored to the unique characteristics of each patient [6]. This study aimed to assess the surgical management, perioperative morbidity, and oncologic outcomes associated with urinary tract resection and repair performed during CRS/HIPEC in patients with carcinomatosis.

## 2. Materials and Methods

After obtaining approval from the institutional review board, 550 consecutive patients aged 18 years or older who underwent CRS/HIPEC procedures aimed at curing carcinomatosis between January 2007 and July 2018 at six university hospitals were included in the study. For all included patients, extra-abdominal metastases were excluded using chest CT and/or PET-CT when clinically indicated.

Urinary tract resection is a surgical procedure involving the excision of part of the urinary tract structure, including the kidneys, ureters, and bladder, primarily performed to manage malignancies, severe trauma, or other pathological conditions affecting these organs. It has been determined that 50 out of these 550 patients had a concomitant curative resection or reconstruction of the bladder, ureter, or kidney during the CRS/HIPEC procedure due to tumor invasion or iatrogenic injury, and these patients’ data were analyzed retrospectively.

Data on patient demographics, American Society of Anesthesiologists (ASA) status, prior neoadjuvant therapy, history of previous surgery, PCI, CC, type of malignancy, type of urologic intervention, nutritional status, co-morbidities, morbidity, mortality, and OS were recorded in each patient.

Severe malnutrition was defined as a weight loss of 20%, moderate malnutrition as a weight loss ranging from 10 to 20%, and mild malnutrition as a weight loss of 10%. Intraoperative assessment of peritoneal disease was performed using the PCI, which evaluates tumor burden by assigning a score from 0 to 3 to each of the 13 anatomical regions within the abdominal cavity. The cumulative PCI score ranges from 0 to 39, reflecting the overall extent of peritoneal carcinomatosis. The total PCI scores were categorized into three groups, including major (scores > 20), intermediate (scores 10–20), and low (scores < 10) tumor burden [21,26,27].

The completeness of cytoreduction was assessed using the Jacquet/Sugarbaker Classification System [26]. Accordingly, CC-0 (no residual macroscopic disease), CC-1 (residual peritoneal deposits < 2.5 mm), CC-2 (residual tumor deposits between 2.5 mm and 2.5 cm), and CC-3 (residual deposits > 2.5 cm or confluent tumors) categories were defined [26]. Complete cytoreduction was considered an eradication of all peritoneal nodules ≥ 2.5 mm in diameter (CC 0–1) [26]. Complications were graded according to the Clavien–Dindo classification, with consideration of a clinically important postoperative complication as a high-grade (III and IV) surgical morbidity. Grade III complications are defined as those requiring surgical, endoscopic, or radiological intervention, while Grade IV complications are life-threatening and require intensive care management [28].

The eligibility criterion for CRS/HIPEC was the histologic or cytologic diagnosis of PM, regardless of the tumor origin. As previously reported, CRS, including a peritonectomy and the resection of the tumor-involved organ(s), was the first crucial step of the radical curative oncologic approach. Non-resectable hepatic and thoracic metastases, involvement of the mesenteric root or major abdomino-pelvic vascular structures, extensive portal pedicule or small bowel invasion, and circumscribed pelvic sidewall infiltration were all considered absolute contraindications to CRS.

The HIPEC procedure was performed simultaneously after CRS using a closed technique with the use of various drugs (mitomycin-C ± cisplatin, doxorubicin, and oxaliplatin), depending on the type of malignancy. However, mitomycin-C was the most commonly used agent in all HIPEC procedures, which was administered at a dose of 40 mg in the dialysis solution for a 90 min perfusion period. During the HIPEC procedure, the target intraperitoneal temperature level was set at 42–42.5 °C [29]. In this multicenter study, the choice of HIPEC procedure was based on the type of malignancy and the discretion of the surgeon and oncologist.

Urinary tract involvement was determined preoperatively in all cases with imaging studies. The use of ureteral stenting was decided at the discretion of the operating surgeon. The kidney was resected in cases with macroscopically significant tumor invasion of the kidney or upper part of the ureter. The ureter and/or part of the bladder were resected when there was a seemingly tumoral adherence or suspicion of being invaded. If the ureter and bladder had been involved in a resection then they were immediately repaired with absorbable sutures. However, in cases where a primary repair was not doable, reconstructive techniques were utilized. All ureteral repairs underwent the placement of J&J (double-J) stents, and a Foley catheter was used in all cases.

### 2.1. Statistical Analysis

Statistical analysis was performed using the statistical package SPSS software (Version 25.0, SPSS Inc., Chicago, IL, USA). For each continuous variable, normality was checked by Kolmogorov–Smirnov and Shapiro–Wilk tests and by histograms. The categorical variables were analyzed by Chi-square or Fisher exact tests, while the numeric variables were analyzed with the Student *t*-test for normally distributed data and the Mann–Whitney U test for non-normally distributed data. *p* < 0.05 was considered statistically significant. OS was defined as the number of years that elapsed between the date of operation and the death as a result of disease (or the last follow-up date). OS was analyzed using the Wald test, and the log-rank test was used to examine the relationship when different parameters were applied. The survival curve was plotted using the standard Kaplan–Meier methodology.

### 2.2. Use of Artificial Intelligence (AI) Tools

An AI-based writing assistance tool was used during manuscript preparation to assist with minor grammatical corrections and improvements in clarity. The tool was not involved in data analysis, interpretation, or content generation.

## 3. Results

Of the total of 550 patients treated with CRS/HIPEC from 2007 to 2018 in the six experienced centers, 50 (9%) patients had undergone resection or a repair of the urinary tract. The mean ± SD age was 51 ± 12.7 years, and there was a female predominance (64%). The most common origins of peritoneal tumor disease were colorectal and ovarian carcinomas (38% + 38%).

Liver metastasis is frequently observed in patients with peritoneal carcinomatosis and typically indicates a high tumor burden. Achieving optimal cytoreduction in such cases often requires extensive upper-abdominal surgery, including liver resection, and is associated with poorer survival outcomes in colorectal cancer patients [30]. However, in our study population, only two patients had liver metastases; therefore, this specific condition was not included in the analysis (Table 1).

The median PCI was 19, and the extent of the peritoneal disease was major (PCI ≥ 20) in 24 (48%) patients. The vast majority of patients [*n* = 25, (50%) + *n* = 20, (40%)] had a CC of 0–1 after cytoreduction (Table 2).

**Figure 1:**
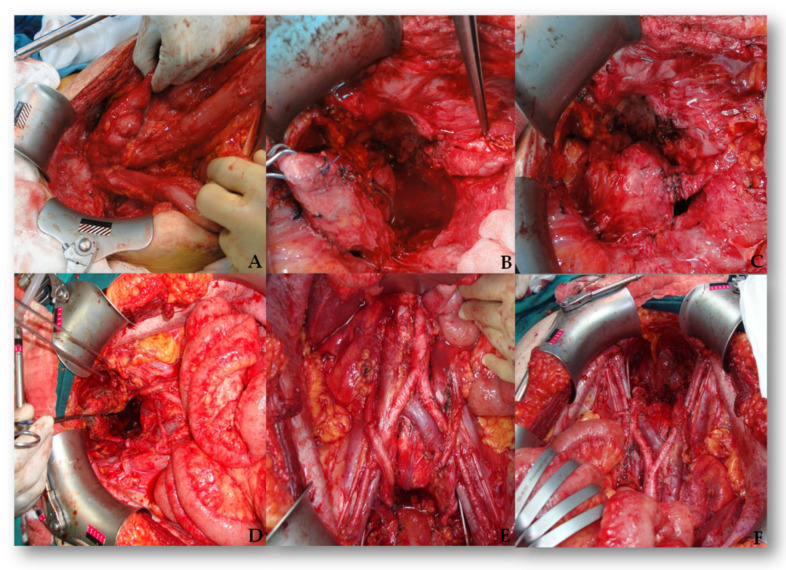
Step by step bladder and ureter repair. (**A**) The ureter injury is identified. (**B**) A cystotomy is made for ureteral reimplantation. (**C**) A double-J stent is inserted. (**D**) The ureter is anastomosed to the bladder mucosa. (**E**) The detrusor muscle is closed over the ureter. (**F**) The completed uretero-neocystostomy.

In total, 56 urologic procedures were performed on 50 patients. Three nephrectomies were performed due to tumor invasion. There were 15 partial cystectomies due to tumor invasion, in addition to 17 injuries to the bladder which had been surgically repaired (Figure 1).

A total of 21 ureteral resections (18 were unilateral and 3 were bilateral) were performed. Among these, 11 were related to iatrogenic injury, recognized intraoperatively by direct visualization of unintended ureter damage, while 10 were due to tumor invasion, determined through gross operative findings and confirmed by histopathological analysis (Table 3).

Nineteen of them were treated with end-to-end anastomoses, and two re-implantations were performed inside the bladder and J&J stents were placed in all of these patients. Six patients died (12%) within 30 days following surgery with multiple organ failure after septic complications, and one of them had urinary leakage.

A total of 25 Clavien–Dindo high-grade (grade 3 or 4) complications occurred in 21 patients (42%), including intra-abdominal collection (*n* = 6), intra-abdominal bleeding (*n* = 4), anastomotic leakage (*n* = 4), wound dehiscence (*n* = 3), intestinal fistula (*n* = 3), respiratory failure (*n* = 2), pneumothorax (*n* = 2), and renal insufficiency (*n* = 1). Re-operation was required in five patients, while all other complications were treated with percutaneous intervention and/or medical treatment. The median postoperative hospital stay was 24 (ranging from 5 to 66) days.

Postoperative (within 30 days) urologic complications due to surgery were observed in 9 (18%) of the 50 patients and all were high-grade (grade 3–4) complications. All of the early urologic complications were urinary leakages that occurred as a uroperitoneal fistula which developed among 21 ureteral anastomoses (30%). The end-to-end ureteral anastomosis was applied in eight cases, while one case was managed with re-implantation inside the bladder during the CRS/HIPEC procedure.

All patients were treated with percutaneous interventions including nephropyelostomy catheters, drainage of the peritoneal collection, and prolonged bladder catheterization.

Nephropyelostomy catheters were used for the treatment of early (within 30 days) urological complications in eight cases. These catheters were removed after the demonstration of no urine leakage in the control antegrade pyelographic images which were performed with a mean interval of 102 ± 74 days (ranging from 39 to 272 days) after insertion.

The univariate analysis findings regarding the factors associated with the development of early urinary complications in patients who underwent CRS/HIPEC are provided in Table 4.

According to the univariate analysis, only one factor, which was a PCI of more than ≥20 (*p* < 0.009), was found to be a significant risk factor for the development of early urinary complications.

The median length of the follow-up was 14 (ranging from 10 to 36) months. A total of 11 of the 50 (22%) patients died at 12 months during the study period. The median OS of the patients was 23 months, and 1-year and 2-year survival rates were 79.8% and 42.9%, respectively. OS time following CRS/HIPEC treatment was similar between patients with and without urologic complications (median OS 23 months vs. 27 months, *p* = 0.683) (Table 5).

**Figure 2:**
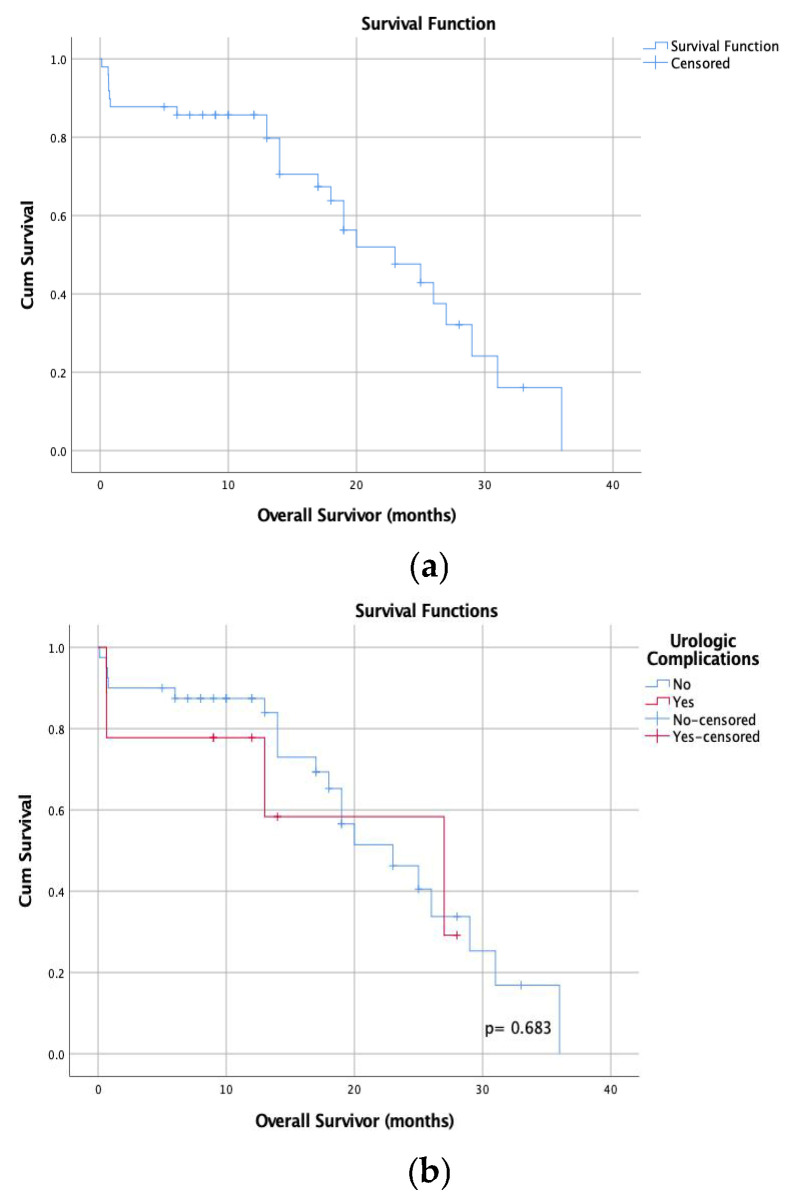
The Kaplan–Meier curves (**a**,**b**). Overall survival of all patients (**a**). No statistical significance was detected in the comparison of overall survival of those patients with and without urinary complications (**b**).

Survival analysis of all patients and a comparison of patients with and without urinary complications are provided in Figure 2.

## 4. Discussion

Patients should be carefully evaluated in the preoperative period, and the surgeons should be prepared in advance for unexpected urologic system involvement. Our findings revealed that a considerable number of patients, around one-tenth, potentially need urologic resection or urologic repair in CRS/HIPEC for PM to obtain optimal cytoreduction. The need for urological resection or repair is directly related to the surgeons’ skillset and the patients’ motivation for achieving complete cytoreduction.

Various studies have reported operative complications related to the urinary tract in the context of CRS/HIPEC procedures [21,22,31,32].

Honore and colleagues demonstrated that in cases of CRS/HIPEC for the treatment of PC, the urinary tract was affected or injured in 8% of instances. Interestingly, despite the complexity of the surgical procedures and the addition of HIPEC, only 12% of associated urologic interventions resulted in the formation of a fistula. A PCI ≥ 20 and the presence of severe preoperative malnutrition have been observed to influence the likelihood of developing a urinary fistula. Consequently, the key takeaway is that the decision to perform CRS with HIPEC should not be ruled out solely based on the application of a potentially risky urinary repair [21]. While the authors reported a low rate of early complications, comprising of fistulas, in all cases, similar to our findings, their analysis did not include the late follow-up. In a study conducted by Votanopoulos et al., it was observed that significant predictors of urological complications included female gender and malnutrition. The occurrence of urinary fistulas was noted in 1.0% of all patients after CRS/HIPEC, with the majority (77.8%) of these cases following urologic procedures. The authors acknowledged a limitation in their data, specifically the absence of information on patients deemed suitable for CRS/HIPEC but who did not undergo HIPEC due to challenges in achieving adequate cytoreduction. This selection bias raised the possibility that certain individuals with urologic involvement might not derive benefits from CRS/HIPEC [22]. In our study, we expanded the inclusion criteria to encompass patients with high cytoreduction scores, enhancing the reliability of our data concerning urological complications and survival outcomes. Lyon et al. conducted a comprehensive analysis of 938 CRS/HIPEC procedures, with 71 of them incorporating 75 urologic interventions. Within the urologic intervention group, only six major complications related to the urinary tract were reported. These comprised two cases of urine leak/fistula, two instances of ureteral stent placement due to obstruction, one vesicovaginal fistula, and one ureteroenteric anastomotic stricture. In contrast, the non-urologic group experienced three major urinary tract complications. The study revealed a higher likelihood of encountering a major urinary tract complication in the urologic intervention group compared to the non-urologic group (8.4% vs. 0.35%, *p* < 0.001) [31]. Our study focuses on comparing the OS outcomes between patients with urinary complications and those without urologic complications. To ensure a thorough investigation, we recognize the importance of separately examining major urinary tract complication rates in both urinary and non-urinary intervention groups for future studies. This approach will enhance the integrity and precision of our research findings.

Determining specific risk factors for the occurrence of urinary complications can offer insights for developing preventive strategies. Our analysis highlighted a PCI value greater than 20 as the exclusive prognostic factor for urological complications. According to Leapman et al., univariate analysis demonstrated an association between distal ureteral repair and a PCI exceeding 20 with the development of long-term urologic complications [32]. In our series, the primary urological method for re-establishing ureteric continuity was end-to-end anastomosis. Nevertheless, a specific study suggested re-implantation with uretero-neocystostomy, particularly in the context of CRS with HIPEC. Given the limited scope of this study, involving only 28 patients, validation through investigations with a larger patient cohort is imperative [33].

The significance of cytoreduction in management was underscored by the PRODIGE-7 trial [34]. Our study revealed a concerning aspect, as five out of fifty patients exhibited a suboptimal CC (CC-2), potentially carrying significant implications for the data. Future studies should concentrate on attaining optimal CC to gather more comprehensive data on survival. In light of the PRODIGE-7 trial, the incorporation of oxaliplatin-based HIPEC alongside CRS did not yield a significant impact on OS or relapse-free survival compared to CRS alone. However, it was associated with a higher incidence of postoperative complications at 60 days. Notably, the curative approach to managing peritoneal metastases secondary to colorectal cancer with CRS alone combined with systemic chemotherapy at specialized cancer centers unexpectedly demonstrated efficacy in achieving long-term recurrence-free survival [34]. Several methodological shortcomings have been identified in PRODIGE-7, particularly in the HIPEC protocol and the selection of OS as the primary endpoint. Consequently, its results have not been considered definitive, underscoring the critical need for further research on HIPEC [35]. The use of the mitomycin-C regimen predominantly utilized in our patients has been associated with enhanced survival, particularly among patients with a low tumor burden [36]. These findings align with the favorable long-term outcomes observed in a collaborative observational study from Korea and Japan, where CRS plus mitomycin-c-based HIPEC demonstrated beneficial results [37]. It appears that the HIPEC protocol involving high-dose mitomycin-C (35 mg/m^2^) is the preferred regimen for future clinical investigations. The GECOP-MMC, an ongoing prospective, open-label, randomized, multicenter phase IV clinical trial, seeks to assess the efficacy of HIPEC with high-dose mitomycin-C in preventing peritoneal recurrence in patients with limited peritoneal metastasis from colon cancer (excluding rectal cases) following complete surgical cytoreduction [35]. A retrospective study from a Chinese center found that CRS/HIPEC combined with urinary tract resection and reconstruction was associated with a high incidence of Grade I–II urinary adverse events, which did not affect OS. While this study supports our findings regarding OS, it focused exclusively on patients with urinary tract invasion. Our study includes a larger cohort, spans multiple centers, and incorporates patients with iatrogenic injuries, providing a more comprehensive view of urinary tract involvement in the CRS/HIPEC procedure [38].

This study has several limitations, primarily due to its retrospective cohort design, which may introduce selection bias and confounding factors, making it difficult to establish cause-and-effect relationships. The multicenter design, while enhancing generalizability, introduced variability in surgical approaches, perioperative management, and HIPEC protocols, including differences in chemotherapeutic agents and dosages, which may have influenced clinical outcomes. These discrepancies could confound outcome assessment and complicate direct interpatient comparisons. Decisions regarding preoperative imaging and intraoperative interventions, such as ureteral stenting, were left to the discretion of the operating surgeon, introducing variability in clinical management. Although all patients undergoing ureteral reconstruction received double-J stent placement and Foley catheterization, data on the timing of stent removal were not collected. Data on patients excluded due to unresectable disease (e.g., hepatic or thoracic metastases) were not reported. Co-morbidities were captured via specific clinical variables such as ASA classification and nutritional status; however, a standardized co-morbidity index (e.g., Charlson Comorbidity Index or Barthel Index) was not employed, which may have limited risk stratification. Finally, although overall survival was analyzed, the lack of longitudinal data on postoperative quality of life and functional recovery limits the comprehensiveness of the outcome assessment following CRS/HIPEC. Additionally, the study did not incorporate biomarker-based risk stratification, which is an emerging area with the potential to improve patient selection and predict postoperative outcomes more accurately.

## 5. Conclusions

In conclusion, urinary tract involvement is neither a contraindication for CRS with HIPEC nor an unsalvageable situation in patients with PC undergoing major abdominopelvic surgery. Even though the PCI value is more than 20, and urinary tract repair during procedures may be associated with increased complication rates, expected survival benefits may still be achievable regardless of urinary tract involvement or injury with CRS/HIPEC. Further studies are needed to confirm the impact of urinary tract involvement on long-term survival.

## Figures and Tables

**Table 1:** Patient characteristics.

Characteristic	Value *n* (%)
Gender	
Female	32 (64%)
Male	18 (36%)
Age (year) mean ± SD (range)	51 ± 12.7 (19–72)
BMI (kg/m^2^) mean ± SD (range)	26.2 ± 5.1 (20–43)
Severe malnutrition	5 (10%)
ASA score	
ASA 1	8 (16%)
ASA 2	26 (52%)
ASA 3	16 (32%)
Origin of carcinomatosis	
Colorectal cancer	19 (38%)
Ovarian cancer	19 (38%)
PMP	9 (18%)
Others	3 (6%)
Neoadjuvant chemotherapy	32 (64%)
Liver metastasis	2 (4%)
Prior stoma	2 (4%)
Prophylactic ureteral stent	8 (16%)
Prior surgery	28 (56%)
Hysterectomy/salpingo-oophorectomy	9 (18%)
Appendectomy	4 (8%)
Colectomy	10 (20%)
Cholecystectomy	5 (10%)

SD: Standard Deviation, ASA: American Society of Anesthesiologists, BMI: Body Mass Index, PMP: Pseudomyxoma Peritonei.

**Table 2:** Operative data.

Operative Data	Value *n* (%)
PCI (median) (range)	19 (5–25)
PCI < 20	26 (52%)
PCI ≥ 20	24 (48%)
Surgical resection (*n*)	262
Pelvic peritonectomy	40 (15.3%)
Omentectomy	44 (16.8%)
Cholecystectomy	25 (9.5%)
Small bowel resection	34 (13%)
Splenectomy	25 (9.5%)
Hepatic metastasectomy	5 (2%)
Gastrectomy	5 (2%)
Diaphragmatic stripping	22 (8.4%)
Colorectal resection	47 (18%)
Hysterectomy/salpingo-oophorectomy	8 (3.1%)
Distal pancreatectomy	6 (2.3%)
Partial resection of psoas muscle	1 (0.4%)
Postoperative stoma creation	24 (48%)
Completeness of cytoreduction score	
CC-0	25 (50%)
CC-1	20 (40%)
CC-2	5 (10%)
Number of anastomosis (median) (range)	1 (0–4)
Operative time(min), median (range)	480 (180–710)
Estimated blood loss (mL), median (range)	400 (100–3200)
Peritonectomy (median) (range)	4 (2–4)
Surgical procedures (median) (range)	5 (1–9)
Surgical procedures ≥ 5	27 (54%)

PCI: Peritoneal Cancer Index, CC: Completeness of Cytoreduction Score.

**Table 3:** Urologic procedures. (**A**) Types of urologic procedures; and (**B**) comparison of urologic procedures: tumor invasion versus iatrogenic injury.

(**A**)		
**Organ/Procedure**	**Resection *n* (%)**	**Reconstruction *n* (%)**
Kidney	3 (5.4%)	-
Ureter	21 (37.5%) (18 unilateral and 3 bilateral)	-
Bladder	15 (partial) (26.8%)	17 (30.4%)
(**B**)		
**Organ/Procedure Reason**	**Tumor Invasion *n* (%)**	**Iatrogenic Injury *n* (%)**
Kidney	3 (5.4%)	-
Ureter	10 (17.9%)	11 (19.6%)
Bladder	15 (26.8%)	17 (30.4%)

**Table 4:** Univariate analysis of risk factors for early (postoperative) urinary complications (within 30 days).

	Urinary Complications		
	Yes (*n* = 9)	No (*n* = 41)	*p* Value
Age (year), mean ± SD	51.3 ± 15.6	50.7 ± 11.6	0.655
BMI (kg/m^2^), mean ± SD	26 ± 2.2	25.4 ± 4.4	0.766
Number of anastomosis, median (range)	2 (0–3)	1 (0–4)	0.426
Estimated blood loss (mL), median (range)	800 (0–1600)	400 (0–3200)	0.619
Peritonectomy, median (range)	4 (2–4)	4 (2–4)	0.661
Operation duration, median (range)	450 (300–600)	480 (180–710)	0.766
Postoperative stoma presence, *n* (%)	5 (55%)	19 (46%)	0.721
Urology consultation, *n* (%)	0	6 (14%)	0.576
Gender (F/M), *n*	6/3	26/15	1
PCI ≥ 20, n (%)	8 (89%)	16 (39%)	**0.009**
Number of surgery ≥ 5, *n* (%)	6 (66%)	21 (51%)	0.479
Etiology, *n* (%)			
Colorectal cancer	6 (66%)	13 (31%)	0.114
Ovarian cancer	2 (22%)	17 (41%)	0.485
PMP	0	9 (22%)	0.283
Others	1 (11%)	2 (4.9%)	0.949
CC-0, *n* (%)	7 (77.8%)	18 (43.9%)	0.138
ASA ≥ 3, *n* (%)	2 (22.2%)	13 (31.7%)	0.705
Severe malnutrition, *n* (%)	1 (11.1%)	4 (9.8%)	1
Prior surgery, *n* (%)	4 (44.4%)	15 (36.5%)	0.713
Prophylactic ureteral stent, *n* (%)	2 (22.2%)	6 (14.6%)	0.623
Hospital stay (days), median (range)	24 (15–66)	21(10–50)	0.112

SD: Standard Deviation, ASA: American Society of Anesthesiologists, BMI: Body Mass Index, PMP: Pseudomyxoma Peritonei, PCI: Peritoneal Cancer Index, CC: Completeness of Cytoreduction Score.

**Table 5:** The overall survival comparison between patients with or without urologic complications.

	Survival Time (Months)				Survival Rate (%)			
	Estimate Median	Std. Error	95% CI		1-Year Survival	2-Year Survival	3-Year Survival	*p* Value
			LB	UB				
**Total**	23	4.1	14.9	31.1	79.8	42.9	0.0	-
**Urinary complications**								
Yes	23	3.8	15.6	30.5	77.8	29.2	0.0	0.683
No	27	4.0	5.5	48.5	83.9	40.5	16.9	

CI: Confidence Interval, LB: Lower Bound, UB: Upper Bound.

## Data Availability

The data presented in this study are available on request from the corresponding author due to ethical restrictions.
